# Number and type of guideline implementation tools varies by guideline, clinical condition, country of origin, and type of developer organization: content analysis of guidelines

**DOI:** 10.1186/s13012-017-0668-7

**Published:** 2017-11-15

**Authors:** Laurel Liang, Jhoni Abi Safi, Anna R. Gagliardi, members of the Guidelines International Network Implementation Working Group

**Affiliations:** 1 Toronto General Hospital Research Institute, 200 Elizabeth Street, Toronto, M5G2C4 Canada; 2 Envisol, 2 - 4 Rue Hector Belioz, 38110 La Tour du Pin, France

**Keywords:** Guidelines, Implementation, Guideline implementation tools, Content analysis

## Abstract

**Background:**

Guideline implementation tools (GI tools) can improve clinician behavior and patient outcomes. Analyses of guidelines published before 2010 found that many did not offer GI tools. Since 2010 standards, frameworks and instructions for GI tools have emerged. This study analyzed the number and types of GI tools offered by guidelines published in 2010 or later.

**Methods:**

Content analysis and a published GI tool framework were used to categorize GI tools by condition, country, and type of organization. English-language guidelines on arthritis, asthma, colorectal cancer, depression, diabetes, heart failure, and stroke management were identified in the National Guideline Clearinghouse. Screening and data extraction were in triplicate. Findings were reported with summary statistics.

**Results:**

Eighty-five (67.5%) of 126 eligible guidelines published between 2010 and 2017 offered one or more of a total of 464 GI tools. The mean number of GI tools per guideline was 5.5 (median 4.0, range 1 to 28) and increased over time. The majority of GI tools were for clinicians (239, 51.5%), few were for patients (113, 24.4%), and fewer still were to support implementation (66, 14.3%) or evaluation (46, 9.9%). Most clinician GI tools were guideline summaries (116, 48.5%), and most patient GI tools were condition-specific information (92, 81.4%). Government agencies (patient 23.5%, clinician 28.9%, implementation 24.1%, evaluation 23.5%) and developers in the UK (patient 18.5%, clinician 25.2%, implementation 27.2%, evaluation 29.1%) were more likely to generate guidelines that offered all four types of GI tools. Professional societies were more likely to generate guidelines that included clinician GI tools.

**Conclusions:**

Many guidelines do not include any GI tools, or a variety of GI tools for different stakeholders that may be more likely to prompt guideline uptake (point-of-care forms or checklists for clinicians, decision-making or self-management tools for patients, implementation and evaluation tools for managers and policy-makers). While this may vary by country and type of organization, and suggests that developers could improve the range of GI tools they develop, further research is needed to identify determinants and potential solutions. Research is also needed to examine the cost-effectiveness of various types of GI tools so that developers know where to direct their efforts and scarce resources.

## Background

Clinical guidelines synthesize scientific evidence on given conditions, diseases, procedures, or therapies to inform health care policy, planning, delivery, evaluation, and quality improvement [1]. Over the last three decades, a plethora of research has shown that, despite their proven benefits [2], guidelines are widely under-used, leading to suboptimal health services and poor patient outcomes [3–5]. The estimated cost of developing a guideline is CAN $100,000 to $1 million [6]. Therefore, on a worldwide basis, scarce resources are being invested in developing guidelines that are not achieving the benefit of which they are intended.

A multitude of determinants, including guideline, clinician, patient, organization, and system level enablers and barriers, influence whether and how guidelines are used [7, 8]. To date, implementation research has largely focused on interventions targeting clinician determinants with limited and inconsistent impact on health care delivery or patient outcomes [9–11]. Far less research has focused on improving the content and format of guidelines to facilitate their adoption, a concept referred to as implementability [12]. Surveys and interviews with clinicians revealed they were aware of and agreed with the guidelines but desired guidance and support to help implement them [13, 14], and 30 guideline developers from seven countries expressed a demand for guideline implementation tools among their users [15]. Research shows that guidelines featuring implementation tools such as quick reference summaries for clinicians, educational material for patients, or indicators or benchmarks for performance measurement are used more often than guidelines lacking such content [16, 17]. Hence, the development and dissemination of guideline implementation tools (GI tools) represent an important way to improve the likelihood of guideline uptake.

Advocates have recommended that guidelines be accompanied by GI tools to support patient-clinician communication and clinical decision-making [18, 19]. Standards and guides for guideline development also recommend that guidelines include GI tools [20–23]. A 2016 Cochrane systematic review confirmed that GI tools developed and disseminated by developers with their guidelines influenced clinician behavior and patient outcomes [24]. To assist guideline developers in generating GI tools, defined as any information in or with guidelines that supports their use, Gagliardi et al. used mixed methods approaches to generate a framework of types of GI tools for different target users and purposes [16], criteria for GI tool content and format based on international consensus [25], and practical considerations for developing GI tools based on the reported experiences of 26 GI tool developers in nine countries [26].

Analyses of guidelines showed that many did not offer GI tools. Among the 20 guidelines published prior to 2010 on the management of diabetes, hypertension, leg ulcers, and heart failure, 45% mentioned the need to actively promote guideline use, but none thoroughly described how to do so, and few offered GI tools for patients or clinicians [16]. A review of 20 studies that used the AGREE instrument to evaluate the quality of 137 guidelines published from 2008 to 2013 found that the applicability domain, pertaining to GI tools, scored lower than all other domains and had not improved significantly compared with the applicability of guidelines published in 2007 or earlier [27]. That study was based on an analysis of secondary data and, hence, did not describe the characteristics of GI tools included in guidelines. Such knowledge could provide insight into the types of organizations and guidelines that offer GI tools, and identify the types of GI tools that more routinely accompany guidelines and those not commonly available, which could inform future GI tool production by guideline developers. The purpose of this study was to describe the number and types of GI tools included in or with guidelines and explore guideline developer and guideline characteristics associated with GI tools. Given the paucity of GI tools revealed in our prior research [16, 27] and subsequently published standards and guides promoting and supporting the development of GI tools [16, 20–23, 25, 26], we hypothesized that the number and type of GI tools would have increased over time.

## Methods

### Approach

Guidelines produced by various types of developers on a variety of clinical topics in different countries were analyzed to identify and describe the GI tools they offered. Content analysis of guidelines and GI tools was employed. Manifest content analysis was used to examine guidelines for the presence of GI tools [28]. This is a method that qualitatively and/or quantitatively describes explicit content as reported in written, verbal, or visual communication, without an interpretation of its underlying meaning. Directed/deductive and summative content analysis techniques were employed to categorize GI tools according to the existing framework of GI tools [16] (directed/deductive) and to enumerate the number and types of GI tools overall and across different types of guidelines [29] (summative). Ethics review and approval was not required because guidelines were publicly available.

### Sampling

Guideline topics were chosen to reflect a range of common chronic and acute conditions that are managed in a variety of health care settings and affect both men and women worldwide. These included English-language guidelines on the overall general management (most often including diagnosis, treatment, and follow-up care, but sometimes also prevention and screening) of arthritis, asthma, colorectal cancer, depression, diabetes (not gestational), heart failure and stroke; guidelines on very specific topics, for example, use of a particular assay for diagnosis, or on rare forms of disease were not eligible. Guidelines published in 2010 or later were included since prior research had largely examined guidelines published prior to 2010 [16, 27] and because the most recent version of the AGREE instrument published in 2010 provided developers with an expanded and more detailed description of types of GI tools (i.e., these may include a summary document, a quick reference guide, educational tools, results from a pilot test, patient leaflets, or computer support) compared with the earlier version published in 2001 (i.e., the guideline is supported with tools for application) [20]. The definition of GI tools was expanded to include any self-contained informational or interactive print or electronic resources in the guideline document or accompanying documents, websites, or applications; instructional information relevant to implementation conveyed in a paragraph or section of the guideline was not considered a GI tool [16].

### Searching and screening

Guidelines were identified in the National Guideline Clearinghouse, a comprehensive, publicly available inventory of international guidelines maintained by the Agency for Healthcare Research and Quality (www.guideline.gov). A research assistant (JAS) searched for guidelines using the name of the condition, for example, arthritis, in both the browse and search features on the National Guideline Clearinghouse website, which identified all general or specific guidelines related to the condition of interest, and compiled a list of all guidelines on those topics published in 2010 or later. ARG and JAS independently reviewed the list and selected eligible guidelines according to sampling criteria. The full text of eligible guidelines and accompanying GI tools were retrieved from the websites of corresponding guideline developers. Searching, screening, and acquiring full-text items were conducted in July 2015. In June 2017, another research assistant (LL) visited developer websites to update searches and independently verify whether all guidelines considered eligible in July 2015 and their GI tools were still available and to independently extract and summarize data. This step identified the most recent versions of guidelines and GI tools originally included.

### Data collection

A data extraction form was developed to collect information on guideline web address, country of development, clinical condition, type of developer (based on data, subsequently categorized into professional society, government agency, disease-specific foundation, non-profit agency, academic institution, or independent expert panel), presence of GI tools (yes/no), GI tool web address, and type of GI tool. GI tools were categorized based on a modified version of the previously published GI tool framework [16]. Table 1 lists the categories and types of GI tools along with descriptions that were used to categorize GI tools. As a pilot test in 2015, ARG and JAS independently analyzed the content of three guidelines and accompanying GI tools and compared and discussed their work to standardize coding and refine the data extraction form. JAS proceeded to analyze the content of all guidelines and GI tools. In 2017, discrepancies in data extraction between LL and JAS were jointly reviewed and resolved by LL and ARG.

**Table 1:** Framework of types of GI tools

Category	Type	Description
Patient support	Information	Print or electronic information about the condition, management options, or additional sources of information
	Guideline summary	Short versions of guidelines designed for patients and care partners
	Self-management support	Resources such as charts, templates, and action plans that can be used by patients to better manage their disease and daily activities
Clinician support	Guideline summary	Short versions of guidelines for clinicians in print or electronic format including pocket cards, summaries, or applications
	Algorithm	Flowcharts or clinical pathways that provide step-by-step guidance for patient management
	Form or checklist	Print or electronic documents to be completed by clinicians for documentation in patient medical records
Implementation support	Training material	Resources to support educational meetings or self-directed learning such as powerpoint presentations or study modules
	Resources	Human, infrastructure or funding resources, or instructions or processes needed for guideline implementation
Evaluation support	Audit tools	Guidelines or manuals to support the evaluation of guideline-compliant practice before and after guideline implementation
	Measures	Quality indicators or performance measures by which to assess compliance with guideline recommendations

### Data analysis

As described above, directed/deductive content analysis was independently applied across three coders to describe guidelines and GI tools. Summary statistics were used to describe guidelines (by number, condition, country, type of organization) and GI tools (by number, category, type of GI tools) and to report their frequency by condition, country, and organization. Exploratory tests of association between GI tools and developer or guideline characteristics were not performed due to small numbers in subgroups.

## Results

### Guideline characteristics

**Fig. 1:**
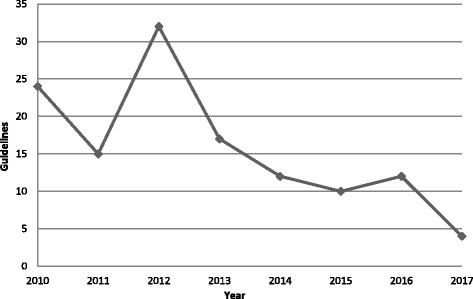
Number of included guidelines published by year

A total of 126 guidelines published from February 2010 to March 2017 were eligible (Table 2). The number of guidelines published per year peaked at 32 in 2012 and subsequently declined on a yearly basis (Fig. 1). The most common conditions addressed by guidelines were heart failure (37, 29.4%), diabetes (29, 23.0%), and stroke (21, 16.7%). The majority of guidelines were produced by organizations based in the USA (76, 60.3%) and UK (27, 21.4%). They were largely produced by professional societies (61, 48.4%), government agencies (28, 22.2%), and disease-specific foundations (17, 13.5%).

**Table 2:** Characteristics of included guidelines

Characteristic	Condition (*n*, %)							Guidelines (*n*, %)
	Arthritis	Asthma	Colorectal cancer	Depression	Diabetes	Heart failure	Stroke	
Organization								
Professional society	7 (46.7)	4 (50.0)	6 (75.0)	2 (25.0)	18 (62.1)	14 (37.8)	10 (47.6)	61 (48.4)
Government agency	6 (40.0)	1 (12.5)	2 (25.0)	3 (37.5)	7 (24.1)	5 (13.5)	4 (19.0)	28 (22.2)
Disease foundation	–	–	–	–	–	14 (37.8)	3 (14.3)	17 (13.5)
Non-profit agency	–	2 (25.0)	–	1 (12.5)	3 (10.3)	–	3 (14.3)	9 (7.1)
Academic institution	–	1 (12.5)	–	2 (25.0)	1 (3.4)	4 (10.8)	1 (4.8)	9 (7.1)
Expert panel	2 (13.3)	–	–	–	–	–	–	2 (1.6)
Country								
United States	7 (46.7)	4 (50.0)	6 (75.0)	4 (50.0)	19 (65.5)	27 (73.0)	9 (42.9)	76 (60.3)
United Kingdom	5 (33.3)	3 (37.5)	1 (12.5)	2 (25.0)	5 (17.2)	6 (16.2)	5 (23.8)	27 (21.4)
Canada	2 (13.3)	–	1 (12.5)	–	3 (10.3)	–	3 (14.3)	9 (7.1)
Australia	–	–	–	1 (12.5)	1 (3.4)	–	3 (14.3)	5 (4.0)
International group	–	1 (12.5)	–	–	–	1 (2.7)	1 (4.8)	3 (2.4)
Finland	–	–	–	–	1 (3.4)	2 (5.4)	–	3 (2.4)
Spain	1 (6.7)	–	–	–	–	–	–	1 (0.8)
Singapore	–	–	–	1 (12.5)	–	–	–	1 (0.8)
Brazil	–	–	–	–	–	1 (2.7)	–	1 (0.8)
Total	15 (11.9)	8 (6.3)	8 (6.3)	8 (6.3)	29 (23.0)	37 (29.4)	21 (16.7)	126 (100.0)

### Guidelines with GI tools

**Fig. 2:**
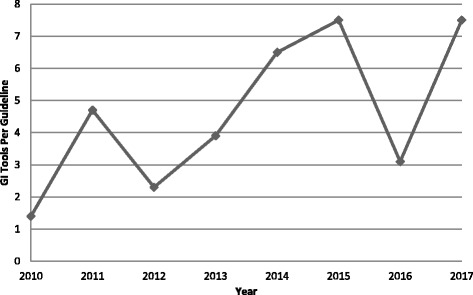
Mean GI tools per included guidelines published by year

Overall, 85 (67.5%) of the 126 guidelines were included or were accompanied by one or more GI tools (Table 3). Guidelines with GI tools were more frequent by condition for asthma (8/8, 100.0%), by country for the UK (26/27, 96.3%), and by type of organization for government agencies (27/28, 96.4%). The mean number of GI tools per guideline increased in 2011 before dropping in 2012, then steadily increased before another drop in 2016, and again increased in 2017 (Fig. 2).

**Table 3:** GI tools by guideline condition, country and organization

Guideline characteristic (*n* guidelines)	One or more GI tools (*n*, % of guidelines)		GI tools (*n*,% of total GI tools)	GI tools per guideline with one or more GI tools			
	Yes	No		Mean	Median	Min	Max
Condition							
Heart failure (37)	19 (51.5)	18 (48.6)	88 (19.0)	4.6	4.0	1.0	11.0
Diabetes (29)	20 (69.0)	9 (31.0)	130 (28.0)	6.5	4.0	1.0	25.0
Stroke (21)	17 (81.0)	4 (19.0)	100 (21.6)	5.9	4.0	1.0	26.0
Arthritis (15)	12 (80.0)	3 (20.0)	35 (7.5)	2.9	3.0	1.0	7.0
Asthma (8)	8 (100.0)	0 (0.0)	50 (10.8)	6.3	4.0	1.0	28.0
Colorectal cancer (8)	3 (37.5)	5 (62.5)	19 (4.1)	6.3	1.0	1.0	17.0
Depression (8)	6 (75.0)	2 (25.0)	42 (9.1)	7.0	7.5	1.0	13.0
Country							
United States (76)	44 (57.9)	32 (42.1)	199 (42.9)	4.5	2.5	1.0	28.0
United Kingdom (27)	26 (96.3)	1 (3.7)	151 (32.5)	5.8	4.5	3.0	17.0
Canada (9)	7 (77.8)	2 (22.2)	90 (19.4)	12.9	8.0	2.0	26.0
Australia (5)	4 (80.0)	1 (20.0)	11 (2.4)	2.8	1.0	1.0	8.0
International group (3)	2 (66.7)	1 (33.3)	8 (1.7)	4.0	4.0	4.0	4.0
Finland (3)	0 (0.0)	3 (100.0)	0 (0.0)	0.0	0.0	0.0	0.0
Spain (1)	0 (0.0)	1 (100.0)	0 (0.0)	0.0	0.0	0.0	0.0
Singapore (1)	1 (100.0)	0 (0.0)	3 (0.6)	3.0	3.0	3.0	3.0
Brazil (1)	1 (100.0)	0 (0.0)	2 (0.4)	2.0	2.0	2.0	2.0
Organization							
Professional society (61)	38 (62.3)	23 (37.7)	135 (29.1)	3.6	2.0	1.0	25.0
Government agency (28)	27 (96.4)	1 (3.6)	166 (35.8)	6.1	5.0	2.0	19.0
Disease foundation (17)	5 (29.4)	12 (70.6)	63 (13.6)	12.6	9.0	1.0	26.0
Non-profit agency (9)	7 (77.8)	2 (22.2)	33 (7.1)	4.7	2.0	1.0	15.0
Academic institution (9)	7 (77.8)	2 (22.2)	66 (14.2)	9.4	7.0	1.0	28.0
International group (2)	1 (50.0)	1 (50.0)	1 (0.2)	1.0	1.0	1.0	1.0
Overall (126)	85 (67.5)	41 (32.5)	464 (100.0)	5.5	4.0	1.0	28.0

### GI tools per guideline

A total of 464 GI tools were identified in or with 85 of the 126 included guidelines (Table 3). The number of GI tools was highest by condition for diabetes guidelines (130, 28.0%), by country for the USA (199, 42.9%), and by organization for government agencies (166, 35.8%). Overall, the mean number of GI tools per guideline was 5.5 (median 4.0, range 1 to 28). The mean number of GI tools per guideline was highest by condition for depression at 7.0 (median 7.5, range 1 to 13), by country for Canada at 12.9 (median 8.0, range 2 to 26), and by organization for disease-specific foundation at 12.6 (median 9.0, range 1 to 26).

### GI tool characteristics

Categories of GI tools by guideline characteristics are summarized in Table 4. The proportion of GI tools reflects the total number of guidelines by condition, country, and organization. Overall, of the 464 GI tools, the majority were to inform and support decision-making among clinicians (239, 51.5%) followed by informing and supporting self-management among patients (113, 24.4%), supporting guideline implementation (66, 14.3%), or supporting evaluation of guideline-concordant care (46, 9.9%). GI tools by condition, country, and organization largely followed this pattern. Exceptions were a fairly even split across the four categories of GI tools for guidelines developed in the UK (patient 18.5%, clinician 25.2%, implementation 27.2%, evaluation 29.1%) or by government agencies (patient 23.5%, clinician 28.9%, implementation 24.1%, evaluation 23.5%). Professional societies generated a high proportion of GI tools for clinicians (97, 71.9%) compared with other types of GI tools.

**Table 4:** Categories of GI tools by guideline characteristic

Guideline characteristic (*n* GI tools)	GI tool category (*n*, %)			
	Patient	Clinician	Implementation	Evaluation
Condition				
Diabetes (130)	37 (28.5)	64 (49.2)	20 (15.4)	9 (6.9)
Stroke (100)	26 (26.0)	63 (63.0)	8 (8.0)	3 (3.0)
Heart failure (88)	15 (17.0)	48 (54.5)	14 (15.9)	11 (12.5)
Asthma (50)	15 (30.0)	30 (60.0)	4 (8.0)	1 (2.0)
Arthritis (35)	12 (34.4)	13 (37.1)	6 (17.1)	4 (11.4)
Depression (42)	5 (11.9)	20 (47.6)	10 (23.8)	7 (16.7)
Colorectal cancer (19)	3 (15.8)	1 (5.3)	4 (21.1)	11 (57.9)
Country				
United States (199)	62 (31.2)	121 (60.8)	16 (8.0)	0 (0.0)
United Kingdom (151)	28 (18.5)	38 (25.2)	41 (27.2)	44 (29.1)
Canada (90)	21 (23.3)	60 (66.7)	8 (8.9)	1 (1.1)
Australia (11)	1 (9.1)	9 (81.8)	0 (0.0)	1 (9.1)
International group (8)	1 (12.5)	7 (87.5)	0 (0.0)	0 (0.0)
Singapore (3)	0 (0.0)	2 (66.7)	1 (33.3)	0 (0.0)
Brazil (2)	0 (0.0)	2 (100.0)	0 (0.0)	0 (0.0)
Finland (0)	0 (0.0)	0 (0.0)	0 (0.0)	0 (0.0)
Spain (0)	0 (0.0)	0 (0.0)	0 (0.0)	0 (0.0)
Organization				
Government agency (166)	39 (23.5)	48 (28.9)	40 (24.1)	39 (23.5)
Professional society (135)	23 (17.0)	97 (71.9)	13 (9.6)	2 (1.5)
Academic institution (66)	29 (46.0)	31 (52.6)	6 (9.5)	0 (0.0)
Disease foundation (63)	18 (27.3)	37 (56.1)	4 (6.1)	4 (6.1)
Non-profit agency (33)	4 (12.1)	25 (75.8)	3 (9.1)	1 (3.0)
International group (1)	0 (0.0)	1 (100.0)	0 (0.0)	0 (0.0)
Overall (464)	113 (24.4)	239 (51.5)	66 (14.2)	46 (9.9)

The proportion of GI tools reflects the total number of guidelines by condition, country, and organization

Table 5 summarizes the guideline characteristics within categories of GI tools. In contrast to Table 4, the proportion of GI tools reflects the total number of GI tools in each category (patient, clinician, implementation, evaluation).

**Table 5:** Guideline characteristics within GI tool categories

Guideline characteristic (*n* GI tools)	GI tool category (*n*, %)			
	Patient	Clinician	Implementation	Evaluation
Overall (464)	113 (24.4)	239 (51.5)	66 (14.2)	46 (9.9)
Condition				
Diabetes	37 (32.7)	64 (26.8)	20 (30.3)	9 (19.6)
Stroke	26 (23.0)	63 (26.4)	8 (12.1)	3 (6.5)
Heart failure	15 (13.3)	48 (20.1)	14 (21.2)	11 (23.9)
Asthma	15 (13.3)	30 (12.6)	4 (6.1)	1 (2.2)
Arthritis	12 (10.6)	13 (5.4)	6 (9.1)	4 (8.7)
Depression	5 (4.4)	20 (8.4)	10 (15.2)	7 (15.2)
Colorectal cancer	3 (2.7)	1 (0.4)	4 (6.1)	11 (23.9)
Country				
United States	62 (54.9)	121 (50.6)	16 (24.2)	0 (0.0)
United Kingdom	28 (24.8)	38 (15.9)	41 (62.1)	44 (95.7)
Canada	21 (18.6)	60 (25.1)	8 (12.1)	1 (2.2)
Australia	1 (0.9)	9 (3.8)	0 (0.0)	1 (2.2)
International group	1 (0.9)	7 (2.9)	0 (0.0)	0 (0.0)
Singapore	0 (0.0)	2 (0.8)	1 (1.5)	0 (0.0)
Brazil	0 (0.0)	2 (0.8)	0 (0.0)	0 (0.0)
Finland	0 (0.0)	0 (0.0)	0 (0.0)	0 (0.0)
Spain	0 (0.0)	0 (0.0)	0 (0.0)	0 (0.0)
Organization				
Government agency	39 (34.5)	48 (20.1)	40 (60.6)	39 (84.8)
Professional society	23 (20.4)	97 (40.6)	13 (19.7)	2 (4.3)
Disease foundation	18 (15.9)	37 (15.5)	4 (6.1)	4 (8.7)
Academic institution	29 (25.7)	31 (13.0)	6 (9.1)	0 (0.0)
Non-profit agency	4 (3.5)	25 (10.5)	3 (4.5)	1 (2.2)
International group	0 (0.0)	1 (0.4)	0 (0.0)	0 (0.0)

In contrast to Table 4, the proportion of GI tools reflects the total number of GI tools in each category (patient, clinician, implementation, evaluation)

#### Patient GI tools

Among the 113 patient GI tools, the proportion was highest by condition for diabetes (37, 32.7%), by country for the USA (62, 54.9%), and by organization for government agencies (39, 34.5%).

#### Clinician GI tools

Clinician GI tools totaling 239 were more commonly featured by condition for diabetes (64, 26.8%) and stroke (63, 26.4%), by country for the USA (121, 50.6%), and by organization for professional societies (97, 40.6%).

#### Implementation GI tools

Implementation GI tools numbering 66 were predominant by condition for diabetes (20, 30.3%), by country for the UK (41, 62.1%), and by organization for government agencies (40, 60.6%).

#### Evaluation GI tools

Guidelines that more commonly offered 46 evaluation GI tools were by condition for heart failure (11, 23.9%) and colorectal cancer (11, 23.9%), by country the UK (44, 95.7%) and by organization government agencies (39, 84.8%).

Table 6 summarizes the types of GI tools within GI tool categories by guideline characteristics.

**Table 6:** Types of GI tools within GI tool categories by guideline characteristics

Guideline characteristic (*n* GI tools)	GI tool categories and types (*n*, %)													
	Patient				Clinician				Implementation			Evaluation		
	Total	Information	Self-management	Guideline summary	Total	Guideline summary	Algorithm	Form or checklist	Total	Training material	Resources	Total	Audit tool	Measures
Overall	113	92 (81.4)	15 (13.3)	6 (5.3)	239	116 (48.5)	92 (38.5)	31 (13.0)	66	37 (56.1)	29 (43.9)	46	30 (65.2)	16 (34.8)
Condition														
Diabetes	37	31 (83.8)	3 (8.1)	3 (8.1)	64	35 (54.7)	18 (28.1)	11 (17.2)	20	12 (60.0)	8 (40.0)	9	6 (66.7)	3 (33.3)
Stroke	26	23 (88.5)	2 (7.7)	1 (3.8)	63	40 (63.5)	13 (20.6)	10 (15.9)	8	3 (37.5)	5 (62.5)	3	3 (100.0)	0 (0.0)
Heart failure	15	9 (60.0)	4 (26.7)	2 (13.3)	48	5 (10.4)	43 (89.6)	0 (0.0)	14	8 (57.1)	6 (42.9)	11	7 (63.6)	4 (36.4)
Asthma	15	10 (66.7)	5 (33.3)	0 (0.0)	30	22 (73.3)	4 (13.3)	4 (13.3)	4	3 (75.0)	1 (25.0)	1	0 (0.0)	1 (100.0)
Arthritis	12	12 (100.0)	0 (0.0)	0 (0.0)	13	5 (38.5)	8 (61.5)	0 (0.0)	6	1 (16.7)	5 (83.3)	4	2 (50.0)	2 (50.0)
Depression	5	4 (80.0)	1 (20.0)	0 (0.0)	20	9 (45.0)	5 (25.0)	6 (30.0)	10	8 (80.0)	2 (20.0)	7	2 (28.6)	5 (71.4)
Colorectal cancer	3	3 (100.0)	0 (0.0)	0 (0.0)	1	0 (0.0)	1 (100.0)	0 (0.0)	4	2 (50.0)	2 (50.0)	11	10 (90.9)	1 (9.1)
COUNTRY														
United States (199)	62	43 (69.4)	13 (21.0)	6 (9.7)	121	58 (47.9)	48 (39.7)	15 (12.4)	16	15 (93.8)	1 (6.3)	0	0 (0.0)	0 (0.0)
United Kingdom (151)	28	26 (92.9)	2 (7.1)	0 (0.0)	38	6 (15.8)	32 (84.2)	0 (0.0)	41	15 (36.6)	26 (63.4)	44	28 (63.6)	16 (36.4)
Canada (90)	21	21 (100.0)	0 (0.0)	0 (0.0)	60	43 (71.7)	4 (6.7)	13 (21.7)	8	6 (75.0)	2 (25.0)	1	1 (100.0)	0 (0.0)
Australia (11)	1	1 (100.0)	0 (0.0)	0 (0.0)	9	5 (55.6)	2 (22.2)	2 (22.2)	0	0 (0.0)	0 (0.0)	1	1 (100.0)	0 (0.0)
International (8)	1	1 (100.0)	0 (0.0)	0 (0.0)	7	3 (42.9)	4 (57.1)	0 (0.0)	0	0 (0.0)	0 (0.0)	0	0 (0.0)	0 (0.0)
Singapore (3)	0	0 (0.0)	0 (0.0)	0 (0.0)	2	1 (50.0)	0 (0.0)	1 (50.0)	1	1 (100.0)	0 (0.0)	0	0 (0.0)	0 (0.0)
Brazil (2)	0	0 (0.0)	0 (0.0)	0 (0.0)	2	0 (0.0)	2 (100.0)	0 (0.0)	0	0 (0.0)	0 (0.0)	0	0 (0.0)	0 (0.0)
Finland (0)	0	0 (0.0)	0 (0.0)	0 (0.0)	0	0 (0.0)	0 (0.0)	0 (0.0)	0	0 (0.0)	0 (0.0)	0	0 (0.0)	0 (0.0)
Spain (0)	0	0 (0.0)	0 (0.0)	0 (0.0)	0	0 (0.0)	0 (0.0)	0 (0.0)	0	0 (0.0)	0 (0.0)	0	0 (0.0)	0 (0.0)
Organization														
Government (166)	39	35 (89.7)	3 (7.7)	1 (2.6)	48	12 (25.0)	32 (66.7)	4 (8.3)	40	14 (35.0)	26 (65.0)	39	25 (64.1)	14 (35.9)
Professional society (135)	23	15 (65.2)	3 (13.0)	5 (21.7)	97	41 (42.3)	48 (49.5)	8 (8.2)	13	11 (84.6)	2 (15.4)	2	1 (50.0)	1 (50.0)
Disease foundation (66)	18	18 (100.0)	0 (0.0)	0 (0.0)	37	25 (67.6)	3 (8.1)	9 (24.3)	4	3 (75.0)	1 (25.0)	4	3 (75.0)	1 (25.0)
Academic institution (63)	29	21 (72.4)	8 (27.6)	0 (0.0)	31	21 (67.7)	3 (9.7)	7 (22.6)	6	6 (100.0)	0 (0.0)	0	0 (0.0)	0 (0.0)
Non-profit agency (33)	4	3 (75.0)	1 (25.0)	0 (0.0)	25	16 (64.0)	6 (24.0)	3 (12.0)	3	3 (100.0)	0 (0.0)	1	1 (100.0)	0 (0.0)
International group (1)	0	0 (0.0)	0 (0.0)	0 (0.0)	1	1 (100.0)	0 (0.0)	0 (0.0)	0	0 (0.0)	0 (0.0)	0	0 (0.0)	0 (0.0)

#### Patient GI tools

Most patient GI tools were information (92, 81.4%), and few were self-management resources (15, 13.3%) or guideline summaries (6, 5.3%). This pattern was consistent regardless of guideline condition, country, or organization.

#### Clinician GI tools

Among 239 clinician GI tools, the majority were guideline summaries (116, 48.5%), followed by algorithms (92, 38.5%), then forms or checklists (31, 13.0%). A notable difference was a predominance of algorithms in guidelines by condition for heart failure and arthritis, by country for the UK, and by organization for government agencies and professional societies.

#### Implementation GI tools

Implementation GI tools were more frequently training material (37, 56.1%) compared with resource implications (29, 43.9%). However, resource implications were more frequent in guidelines by condition for stroke (5, 62.5%) and arthritis (5, 83.3%), by country for the UK (26, 63.4%), and by organization for government agencies (26, 65.0%).

#### Evaluation GI tools

More evaluation GI tools were audit tools (30, 65.2%) compared with performance measures (16, 34.8%) except for depression guidelines that featured a high proportion of performance measures (5, 71.4%).

## Discussion

This study found that over two thirds of 126 guidelines published from 2010 to 2017 included or were accompanied by one or more GI tools while nearly one third did not. Although the number of guidelines published per year declined after 2012, potentially due to a change in National Guideline Clearinghouse procedures from actively collecting published guidelines (pull) to receiving submissions from guideline authors (push), the mean number of GI tools per guideline increased, with a spike in 2011, perhaps following publication of the 2010 AGREE instrument [20], and another increase subsequent to 2012, perhaps following publication of the 2011 IOM standards [21]. This finding confirms our hypothesis that the number of GI tools would have increased. The majority of 464 GI tools were for clinicians, fewer were for patients, and fewer still were to support implementation or evaluation. GI tools were predominantly guideline summaries for clinicians and, to a lesser degree, condition-specific information summaries for patients. This finding rejects our hypothesis that the number of GI tools of various types would have increased. Guideline condition did not consistently influence the presence, number, or types of GI tools. Guideline developers in the USA and UK more frequently included GI tools in or with their guidelines, though this finding may reflect the preponderance of guidelines from those countries. Guidelines from Canada featured the highest mean number of GI tools per guideline. Government agencies and developers based in the UK were more likely to generate guidelines that offered all four types of GI tools (patient, clinician, implementation, evaluation) while professional societies were more likely to generate guidelines that included clinician GI tools.

Despite evidence of the impact of GI tools on clinician behavior and patient outcomes [24], requests from clinicians for help with implementing guidelines [13, 14], recommendations, and standards that guidelines include GI tools [18–23], and the existence of frameworks and instructions for developing GI tools [16, 25, 26], our study found that one third of guidelines examined did not offer any GI tools, although the mean number of GI tools per guideline increased over time. These findings are comparable to those of other analyses of guidelines for the presence of GI tools. Our previous research found that few guidelines published in 2013 or earlier offered GI tools for patients or clinicians [16, 27]. More recently, a synthesis of 25 studies that evaluated 415 guidelines published between 1992 and 2014 also found that guidelines scored poorly for the presence of GI tools though, similar to our study, there appeared to be a statistically significant improvement over time [30]. This study generated findings unique from prior research. The numbers and types of GI tools varied across included guidelines by conditions, countries, and organizations. Moreover, exploratory analysis based on an existing framework of numerous possible types of GI tools that could be used by different stakeholders [16] revealed that the majority of GI tools were guideline summaries for clinicians.

This analysis of the most prevalent types of GI tools in guidelines reveals numerous opportunities for developers or others to produce GI tools that may better facilitate guideline implementation and uptake. While the 2016 Cochrane systematic review that demonstrated the effectiveness of GI tools focused largely on printed educational GI tools for clinicians [24], other available research suggests that other types of GI tools could also be effective for promoting guideline uptake. In our study, the majority of GI tools were for clinicians but most of those were guideline summaries rather than point-of-care forms or checklists that could more proactively support guideline uptake and be integrated with electronic medical record or computer decision support systems, a strategy that has been found to improve compliance with guideline-recommended care [31]. Fewer GI tools were aimed at patients and, of those, most were informational rather than self-management resources that could more proactively support patient-clinician communication and guideline compliance by patients [32, 33], or decision aids, which have been shown to improve patient-clinician communication, patient knowledge, and value-congruent treatment choices [34]. Similarly, few GI tools were identified for implementation and evaluation that could be used by managers and policy-makers to monitor and improve care according to guideline recommendations.

Given the proven impact of GI tools on guideline uptake and associated outcomes, our research suggests that developers should shift their focus to incorporate a wider range of types of GI tools in or with their guidelines. However, that may be easier said than done. These findings warrant some consideration of why guidelines do not consistently offer GI tools, or GI tools for the different purposes and stakeholders outlined in Table 1. One reason may be that more definitive evidence of their impact, required to convince developers of the need to generate GI tools, was only published fairly recently in 2016 [24]. Another possible explanation is that guideline summaries are the easiest and quickest GI tool to generate since they are based on the same content as the guideline whereas other types of GI tools such as forms or checklists require additional information, formatting, and perhaps even pilot-testing. These additional steps may require specific expertise and skills that guideline developers may lack and costs in addition to those needed to generate guidelines. The issue of resources may explain why government agencies, particularly in single-payer health systems with a vested interest in implementation, evaluation, and quality improvement, appear to develop guidelines offering a range of types of GI tools while professional societies, with potentially fewer resources, prioritized the development of GI tools for use by their member constituents, the clinicians. Our research involving interviews with representatives of 30 guideline developers, including 12 government agencies and 18 professional societies in seven countries, found that most had little to no funding or staff dedicated to implementation [15]. The lack of GI tools for patients is also notable because research shows that guidelines that address patient preferences are more likely to be used [35, 36] and there are many ways to incorporate patient preferences in guidelines [37]. Furthermore, patients have requested greater access to guidelines to support self-management [38] and we generated a framework of types of GI tools that could support different self-management functions [32, 33]. The small number of patient GI tools offered in guidelines may be attributed to limited resources, and perhaps also to the historical role of guidelines that focused on empirical evidence of clinical effectiveness to routinize clinical practice [39]. However, further research is required to establish factors that influence the number and type of GI tools included in or with guidelines by developers. If insufficient resources are the key barrier, then health system funders and research funders may be compelled to provide targeted funding. Another option is for developers to collaborate with researchers or other types of organizations such as disease-specific foundations or patient advocacy groups to generate GI tools.

Strengths of this study that may enhance transferability and validity of its findings include analysis of guidelines representing a wide range of common clinical conditions produced by different types of organizations in various countries, analysis of guidelines produced in the last several years that are still available, analysis of guidelines and GI tools by multiple coders independently, and characterization of GI tools based on an existing framework revealing gaps that may shape the future design and inclusion of GI tools in guidelines [16]. Limitations include the fact that only one source was used to identify guidelines and only English-language guidelines were included; therefore, all guidelines on the clinical topics of interest may not have been identified, resulting in small numbers of guidelines (and GI tools) for some conditions, countries, and types of organization, thus limiting the exploration of determinants of the inclusion of GI tools in guidelines and limiting analyses of associations between the number or type of GI tools and clinical condition, country, or organization. However, the National Guideline Clearinghouse is one of the largest repositories for guidelines produced internationally, providing samples that can be highly representative of guidelines on the topics of interest. GI tools included in or with eligible guidelines could have been missed during data collection and extraction. To overcome this limitation, two research assistants independently searched each eligible guideline, accompanying documents, and the organizational websites for relevant GI tools. Lastly, we did not collect data that enabled definitive analysis of reasons for variations in numbers and types of GI tools across conditions, countries, and organizations and did not assess the quality of the identified GI tools. Both of these aspects were beyond the scope of this study and could be addressed in future research.

Few studies have described the costs of generating guidelines and associated products. Therefore, future research should assess the necessary expertise and resources so that guideline developers can anticipate, acquire, or make a business case and budget for GI tool development. Further research is warranted to evaluate the specific types and characteristics of clinician, patient, implementation, and evaluation GI tools that optimize guideline implementation and associated behavioral and clinical outcomes, including which GI tools are more impactful in print or electronic format. Another outstanding issue is the degree to which GI tools should be evidence-based and rigorously evaluated to establish their effectiveness, which also has implications for the expertise and funds required to develop GI tools. Such knowledge would help developers choose GI tools appropriate for a given guideline and decide where to direct their efforts and resources.

## Conclusions

The study suggests that many guidelines did not include or were not accompanied by any GI tools, or a variety of GI tools for different stakeholders despite evidence of the impact of GI tools on guideline uptake, and the availability of frameworks and guidance for generating GI tools. Developers should consider including GI tools for clinicians that could be integrated with computer decision support systems at the point-of-care, for patients to support decision-making and self-management, and for managers or policy-makers to evaluate and improve care. The variation in numbers and types of GI tools by guideline, condition, country, and organization implies a potential for improvement among many guideline developers. However, research is needed to establish reasons underlying the lack of GI tools to reveal strategies that may be needed to foster GI tool development such as targeted funding from health system and research funders. Research is needed to examine the cost-effectiveness of various types of GI tools so that developers know where to direct their efforts and scarce resources.

## Abbreviations

GI tools: Guideline implementation tools
